# Retraction Note to: Dose-dependent changes in neuroinflammatory and arachidonic acid cascade markers with synaptic marker loss in rat lipopolysaccharide infusion model of neuroinflammation

**DOI:** 10.1186/s12868-017-0360-5

**Published:** 2017-05-09

**Authors:** Matthew Kellom, Mireille Basselin, Vasken L Keleshian, Mei Chen, Stanley I Rapoport, Jagadeesh S Rao

**Affiliations:** Brain Physiology and Metabolism Section, National Institute on Aging, National Institutes of Health, 9000 Rockville Pike, Bldg. 9, 1S-126, Bethesda, MD USA

## Retraction Note to: BMC Neuroscience 2012, 13:50 DOI 10.1186/1471-2202-13-50

This article [1] has been retracted by the editor because author Stanley I Rapoport alerted the editor, and the National Institutes of Health subsequently confirmed, that the data represented by figure 5A and 5C were falsified. Stanley I Rapoport supports this retraction. The other authors have not responded to our correspondence with them about the retraction of their article.
